# Escalated‐dose radiotherapy for unresected locally advanced pancreatic cancer: Patterns of care and survival in the United States

**DOI:** 10.1002/cam4.7434

**Published:** 2024-06-26

**Authors:** Christopher Shi, Brian De, Hop S. Tran Cao, Suyu Liu, Marcus A. Florez, Ramez Kouzy, Adam J. Grippin, Matthew H. G. Katz, Ching‐Wei D. Tzeng, Naruhiko Ikoma, Michael P. Kim, Sunyoung Lee, Jason Willis, Sonal S. Noticewala, Bruce D. Minsky, Grace L. Smith, Emma B. Holliday, Cullen M. Taniguchi, Albert C. Koong, Prajnan Das, Ethan B. Ludmir, Eugene J. Koay

**Affiliations:** ^1^ Department of Gastrointestinal Radiation Oncology The University of Texas MD Anderson Cancer Center Houston Texas USA; ^2^ Department of Surgical Oncology The University of Texas MD Anderson Cancer Center Houston Texas USA; ^3^ Department of Biostatistics The University of Texas MD Anderson Cancer Center Houston Texas USA; ^4^ Department of Gastrointestinal Medical Oncology The University of Texas MD Anderson Cancer Center Houston Texas USA

**Keywords:** adenocarcinoma, chemoradiotherapy, definitive therapy, dose escalation

## Abstract

**Introduction:**

With locally advanced pancreatic cancer (LAPC), uncontrolled local tumor growth frequently leads to mortality. Advancements in radiotherapy (RT) techniques have enabled conformal delivery of escalated‐dose RT (EDR), which may have potential local control and overall survival (OS) benefits based on retrospective and early prospective studies. With evidence for EDR emerging, we characterized the adoption of EDR across the United States and its associated outcomes.

**Methods:**

We searched the National Cancer Database for nonsurgically managed LAPC patients diagnosed between 2004 and 2019. Pancreas‐directed RT with biologically effective doses (BED_10_) ≥39 and ≤70 Gy was labeled conventional‐dose RT (CDR), and BED_10_ >70 and ≤132 Gy was labeled EDR. We identified associations of EDR and OS using logistic and Cox regressions, respectively.

**Results:**

Among the definitive therapy subset (*n* = 54,115) of the entire study cohort (*n* = 91,493), the most common treatments were chemotherapy alone (69%), chemotherapy and radiation (29%), and RT alone (2%). For the radiation therapy subset (*n* = 16,978), use of pancreas‐directed RT remained between 13% and 17% over the study period (*p*_*trend*_ > 0.999). Using multivariable logistic regression, treatment at an academic/research facility (adjusted odds ratio [aOR] 1.46, *p* < 0.001) and treatment between 2016 and 2019 (aOR 2.54, *p* < 0.001) were associated with greater receipt of EDR, whereas use of chemotherapy (aOR 0.60, *p* < 0.001) was associated with less receipt. Median OS estimates for EDR and CDR were 14.5 months and 13.0 months (*p* < 0.0001), respectively. For radiation therapy subset patients with available survival data (*n* = 13,579), multivariable Cox regression correlated EDR (adjusted hazard ratio 0.85, 95% confidence interval 0.80–0.91; *p <* 0.001) with longer OS versus CDR.

**Discussion and Conclusions:**

Utilization of EDR has increased since 2016, but overall utilization of RT for LAPC has remained at less than one in five patients for almost two decades. These real‐world results additionally provide an estimate of effect size of EDR for future prospective trials.

## 1 INTRODUCTION

Locally advanced pancreatic cancer (LAPC) accounts for approximately 80% of pancreatic cancer cases and is associated with a poor prognosis, with median survival of 13–24 months.^1^, ^2^ Mortality for patients with LAPC is driven by both early metastatic spread and uncontrolled local growth; an estimated 30% of patients with LAPC die with local progression alone, and an additional 10%–25% experience both local progression and distant spread before death.^3^, ^4^ Treatments that confer durable local control of primary tumor, including radiation therapy (RT) have been shown to reduce morbidity and potentially prolong survival in patients with LAPC.^5^

While recent advances in systemic therapies have improved distant disease control and overall survival,^6^ conventional‐dose RT (CDR) following upfront gemcitabine‐based chemotherapy may be ineffective for local control of disease.^7^, ^8^ With technological advances over the last 20 years enabling more precise RT delivery, escalated‐dose RT (EDR) represents a safe and promising alternative to CDR.^9^, ^10^, ^11^ Though randomized evidence supporting EDR use is lacking, several institutional series, meta‐analyses, and prospective phase 1–2 studies have demonstrated high rates of local control and favorable toxicity profiles.^12^, ^13^, ^14^, ^15^ The use of EDR following upfront chemotherapy is correspondingly supported for appropriately selected patients with LAPC by consensus guidelines.^8^, ^16^, ^17^

Despite growing evidence of the potential benefits of EDR, the extent to which it has been adopted in the United States over the past two decades is not known. Therefore, we used the National Cancer Database (NCDB), a clinical oncology database representing more than 70% of newly diagnosed cancers in the United States, to develop a contemporary assessment of factors associated with the use of EDR for LAPC and survival following its use.

## 2 METHODS

### 2.1 Patient characteristics

This study was reviewed and approved by the Institutional Review Board review at MD Anderson Cancer Center. The NCDB is a U.S. hospital‐based cancer registry, jointly maintained by the American College of Surgeons Commission on Cancer and the American Cancer Society. It collects patient data from >1500 centers across the United States. After approval by our institutional review board, we identified the population of interest from the NCDB. For analysis, we extracted patients diagnosed between 2004 and 2019, inclusive, with an International Classification of Diseases O‐3 code of C25 (*n* = 498,748). We excluded patients if they had neuroendocrine tumors, non‐adenocarcinoma neoplasms, noninvasive disease, and/or American Joint Committee on Cancer (AJCC) TNM staging that was incomplete or missing. We also excluded patients with T0–T1, N2, and/or M1 disease; individuals with N2 disease were excluded because they are less likely to be candidates for dose escalation, given the proximity of critical structures such as the duodenum. Additionally, we excluded those for whom pancreas resection was recommended and/or performed as well as those with unknown pancreas resection status.

### 2.2 Treatment variables and covariates

Regimens for RT alone, chemotherapy alone, or chemotherapy and radiation (CT + RT) were recorded, and the numbers of chemotherapy agents, if applicable, were noted. The NCDB records radiotherapy doses from up to three phases for a given treatment course: phase I, II, and III, which account for potential boosts and/or cone down treatments. The BED_10_ from phases I, II, and III were summed to provide a total BED_10_ across all three phases. Pancreas‐directed RT with biologically effective doses (BED_10_) >70 and ≤132 Gy were defined as EDR, and BED_10_ between 39 and 70 Gy, inclusive, were labeled as CDR. BED_10_ was calculated using the linear‐quadratic model as:

$${\mathrm{BED}}_{\alpha /\beta }=n^{*}d\;\left(1+d/\left(\alpha /\beta \right)\right)$$

where *n* is the number of fractions, *d* is the dose per fraction, and *α*/*β* is a parameter of tissue sensitivity to fractionation, assumed to be 10 for pancreatic ductal adenocarcinoma.^18^ The lower bound for CDR, BED_10_ 39 Gy, was selected to correspond to 30 Gy in 10 fractions. The lower bound for EDR, BED_10_ 70 Gy, was chosen based on the experience at MD Anderson Cancer Center and other institutions.^10^, ^11^, ^14^, ^19^ Finally, the upper bound for EDR, BED_10_ 132 Gy, was chosen to correlate with 60 Gy in 5 fractions, a plausible, previously described upper limit for hypofractionated regimens.^10^, ^20^ The most common dose and fractionation regimens for CDR and EDR were reported.

Other covariates captured included age at diagnosis, sex, race, ethnicity, primary tumor site, AJCC T and N stages, histologic grade, Charlson‐Deyo comorbidity score, insurance carrier, facility type, facility volume, facility location, patient residential setting, distance to treatment facility, year of diagnosis, TNM staging edition (6th, 7th, or 8th), and use of chemotherapy. To account for multicollinearity and minimize overfitting in the multivariable analysis, variable selection utilized elimination of the variable with the highest variance inflation factor (VIF) until the mean VIF of remaining variables was ≤4; these variables comprised the final model. Other clinically relevant variables such as age at diagnosis were added based on intuitive possible correlations with survival. CA‐19‐9 levels were not available for most patients; these data were not considered for analysis. To study temporal associations, the study period was divided into four groups, each spanning 4 years and roughly correlating with the release of data from seminal trials such as Eastern Cooperative Oncology Group (ECOG) 4201 in 2011 and LAP‐07 in 2016: 2004–2007, 2008–2011, 2012–2015, and 2016–2019.

### 2.3 Analysis of RT utilization and outcomes

Overall RT utilization per year was calculated as a proportion of patients with unresected LAPC and treatment details available who received definitive‐intent treatment. EDR utilization was calculated from the analytic cohort as the proportion of all unresected LAPC patients with complete treatment information receiving definitive pancreas‐directed RT using escalated doses, as previously described. Utilization rates were calculated for each year in the study period. Temporal trends in RT utilization were also stratified by RT technique.

### 2.4 Statistical analysis

All data management and analysis was performed using STATA/BE 17.0 (StataCorp, College Station, TX). To identify differences in cohort composition, two‐sample Wilcoxon rank‐sum tests were utilized for continuous variables and chi‐squared tests were used for categorical variables, respectively. The Cochran‐Armitage and Mann‐Kendall nonparametric trend tests were performed to assess differences in linear and monotonic trends, respectively, by year of diagnosis. Univariate and multivariable logistic regressions were performed to identify associations with receipt of EDR versus CDR. To identify associations with survival, a Cox proportional hazards model was used. The proportional hazards assumptions were evaluated using a chi‐squared test of Schoenfeld residuals; tests of the proportional hazards assumptions for death yielded *p* > 0.05 and thus we failed to reject the null hypothesis that hazards were proportional.

To anticipate concerns regarding immortal time bias, a sensitivity landmark analysis of survival was performed for a subset of patients who received CT + RT and for whom the latency from diagnosis to RT initiation was recorded. The landmark timepoint chosen was at RT initiation to attempt to isolate outcomes following RT. For CT + RT recipients, we limited the time between initiation of chemotherapy and that of RT such that recorded chemotherapy was received 30 or more days before or after RT. With these criteria, we conducted a multivariable Cox regression landmark analysis with the origin at the date of RT initiation. Bonferroni corrections were implemented to establish appropriate threshold *p* values to correct for multiple comparisons.

## 3 RESULTS

**FIGURE 1:**
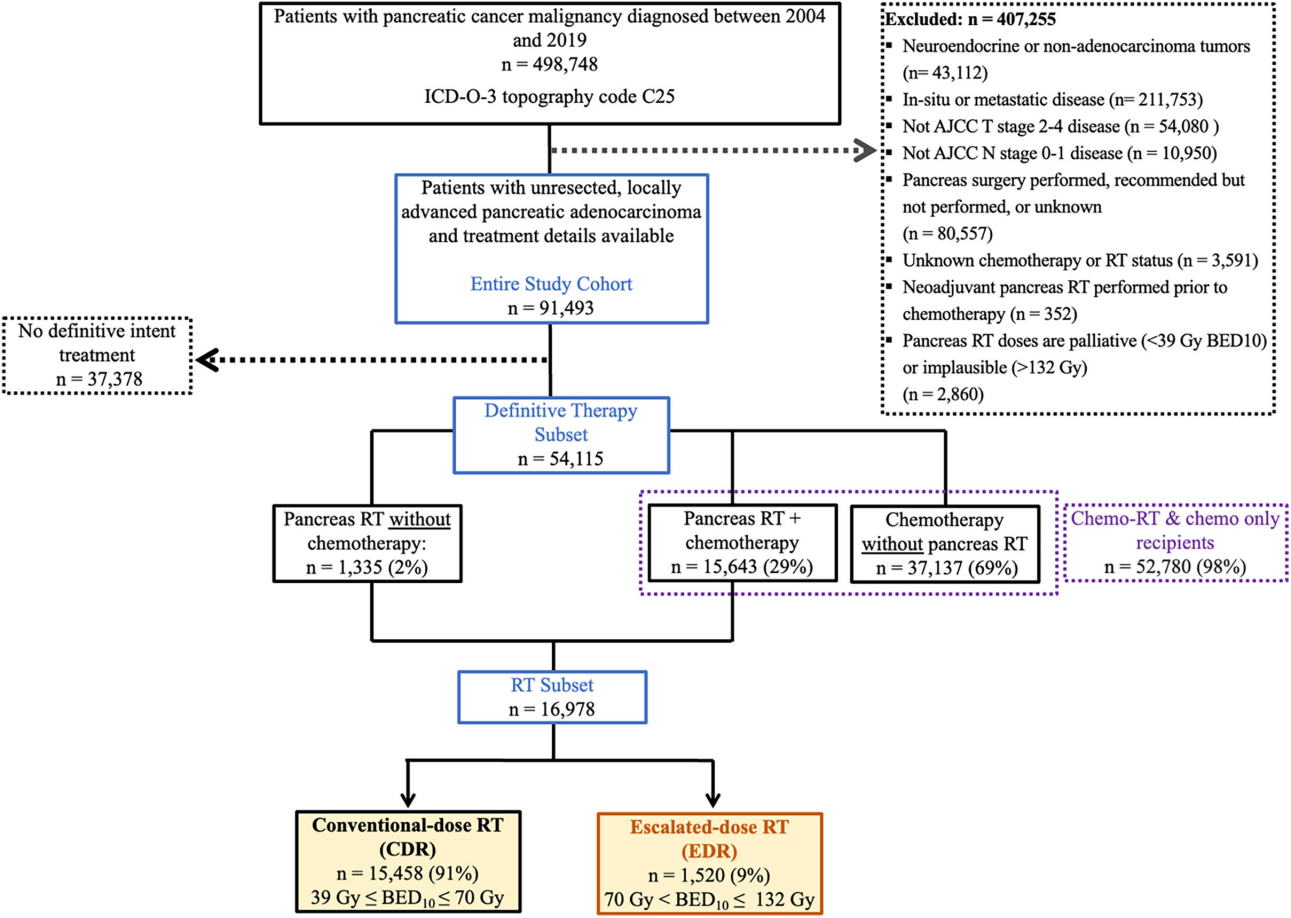
Study inclusion criteria and treatment distribution for patients with unresected pancreatic cancer. AJCC indicates American Joint Committee on Cancer; BED_10_, biologically effective dose assuming an α/β ratio of 10; CDR, conventional‐dose radiotherapy; EDR, escalated‐dose radiotherapy; ICD, International Classification of Diseases; RT, radiotherapy.

The entire study cohort was comprised of 91,943 patients with unresected LAPC (Figure 1). Of the entire study cohort, 37,378 were recorded as having received no definitive‐intent treatment, 10% of whom had contraindications for receipt of chemotherapy because of patient risk factors including advanced age, comorbid conditions, and tumor progression prior to administration. Another 16% did not receive chemotherapy despite having it offered because the patient died or refused treatment. Additionally, 15% of these patients received some type of palliative therapy—surgery, RT, systemic therapy, and/or pain management—and 0.5% received, or were recommended for, but did not receive, immunotherapy. The reason for no definitive‐intent treatment was recorded for 27,015 (72%) of these 37,378 patients: 74% did not receive any treatment; 24% received another therapy besides surgery, chemotherapy, or radiotherapy; and 1% were placed under active surveillance.

Among the definitive therapy subset (*n* = 54,115), the most common treatments were chemotherapy without pancreas‐directed RT (69%), concurrent or adjuvant pancreas‐directed RT with chemotherapy (29%), and pancreas‐directed RT without chemotherapy (2%). Composed of 31% patients in the definitive therapy subset, the radiation therapy subset (*n* = 16,978) received definitive pancreas‐directed RT, 9% of whom received EDR (*n* = 1520). Baseline characteristics for the radiation therapy subset, stratified by RT dose, are displayed in Table 1.

**TABLE 1:** Baseline patient, disease, and treatment characteristics for patients in radiation therapy subset, stratified by receipt of conventional‐dose versus escalated‐dose radiotherapy^a^.

Attribute	Conventional‐dose RT (*n* = 15,458)	Escalated‐dose RT (*n* = 1520)	*p* Value
Median age at diagnosis, y (IQR)	68.0 (60.0, 76.0)	69.0 (62.0, 77.0)	<0.001
Sex			
Male	7905 (51.1%)	768 (50.5%)	0.65
Female	7553 (48.9%)	752 (49.5%)	
Race			
White	12,686 (82.7%)	1277 (84.7%)	0.13
Black	2076 (13.5%)	179 (11.9%)	
Other	583 (3.8%)	51 (3.4%)	
Hispanic ethnicity			
Non‐Hispanic	14,122 (95.9%)	1367 (94.5%)	0.013
Hispanic	601 (4.1%)	79 (5.5%)	
Primary site of tumor			
Body‐tail	2828 (18.3%)	324 (21.3%)	0.011
Head	10,124 (65.5%)	947 (62.3%)	
Other	2506 (16.2%)	249 (16.4%)	
T stage			
T2	3030 (19.6%)	347 (22.8%)	0.003
T3–T4	12,428 (80.4%)	1173 (77.2%)	
N stage			
N0	10,385 (67.2%)	1078 (70.9%)	0.003
N1	5073 (32.8%)	442 (29.1%)	
Charlson‐Deyo comorbidity score			
0–1	14,112 (91.3%)	1372 (90.3%)	0.18
≥ 2	1346 (8.7%)	148 (9.7%)	
Insurance carrier			
No insurance	303 (2.0%)	22 (1.5%)	0.003
Private	5231 (34.3%)	465 (31.0%)	
Medicaid	810 (5.3%)	69 (4.6%)	
Medicare	8679 (56.9%)	911 (60.7%)	
Other government	229 (1.5%)	34 (2.3%)	
Facility type			
Nonacademic/research	8886 (57.8%)	675 (44.7%)	<0.001
Academic/research	6477 (42.2%)	835 (55.3%)	
Facility volume for pancreas‐directed RT patients in 2004–2019, median (IQR)	30.0 (13.0, 64.0)	44.0 (17.0, 117.0)	<0.001
Facility location			
New England	1291 (8.4%)	77 (5.1%)	<0.001
Middle Atlantic	2495 (16.2%)	283 (18.7%)	
South Atlantic	3270 (21.3%)	327 (21.7%)	
East North Central	2920 (19.0%)	258 (17.1%)	
East South Central	989 (6.4%)	75 (5.0%)	
West North Central	1442 (9.4%)	242 (16.0%)	
West South Central	804 (5.2%)	91 (6.0%)	
Mountain	681 (4.4%)	48 (3.2%)	
Pacific	1471 (9.6%)	109 (7.2%)	
Patient residential setting			
Metro	12,432 (83.4%)	1150 (81.3%)	0.041
Urban	2148 (14.4%)	238 (16.8%)	
Rural	329 (2.2%)	27 (1.9%)	
Distance to treatment facility, miles			
0–5	3544 (25.4%)	274 (20.3%)	<0.001
5.1–10	2961 (21.2%)	239 (17.7%)	
10.1–30	4243 (30.4%)	386 (28.6%)	
>30	3213 (23.0%)	451 (33.4%)	
Year of diagnosis			
2004–2007	2917 (18.9%)	210 (13.8%)	<0.001
2008–2011	4114 (26.6%)	342 (22.5%)	
2012–2015	5137 (33.2%)	338 (22.2%)	
2016–2019	3290 (21.3%)	630 (41.4%)	
Use of chemotherapy			
No	1146 (7.4%)	189 (12.4%)	<0.001
Yes	14,312 (92.6%)	1330 (87.6%)	
Median nominal total pancreas‐directed RT dose, Gy (IQR)	45.0 (45.0, 50.4)	55.0 (40.0, 60.0)	<0.001
Median number of pancreas‐directed RT fractions (IQR)	28.0 (25.0, 28.0)	10.0 (5.0, 30.5)	<0.001
Median total pancreas‐directed RT BED, Gy (IQR)	54.8 (52.2, 59.5)	77.1 (72.0, 97.9)	<0.001

*Note*: Analyses comparing treatment cohorts used the Wilcoxon rank‐sum test for continuous variables and chi‐squared test for categorical variables. Percentages may not add to 100% because of rounding.

Abbreviations: BED, biologically effective dose; IQR, interquartile range; RT, radiotherapy.

^a^ Patients without dose information have been excluded.

### 3.1 RT dosing and technique

**FIGURE 2:**
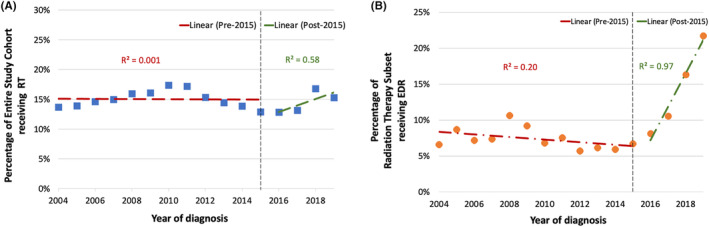
Time series trends in utilization of pancreas‐directed RT and EDR. (A) Percentages of patients in entire study cohort receiving RT and (B) Percentages of patients in radiation therapy subset receiving EDR. EDR indicates escalated‐dose radiotherapy; LAPC, locally advanced pancreatic adenocarcinoma; RT, radiotherapy.

The most frequently utilized dose/fractionation schemes for CDR were 50.4 Gy in 28 fractions (BED_10_ 59.5 Gy; 25%), 45 Gy in 28 fractions (BED_10_ 52.2 Gy; 11%), and 45 Gy in 25 fractions (BED_10_ 53.1 Gy; 10%). The most common EDR regimens were 40 Gy in 5 fractions (BED_10_ 72 Gy; 17%), 59.4 Gy in 33 fractions (BED_10_ 70.09 Gy; 13%), and 50 Gy in 5 fractions (BED_10_ 100 Gy; 13%). Temporal trends in the use of RT are shown in Figure 2. Use of any pancreas‐directed RT grew from 14% in 2004 to a peak of 17% in 2010, declined to a nadir of 13% in 2016, and increased to 15% by 2019. Overall, a statistically significant monotonic trend in the utilization rate of pancreas‐directed RT was not detected (*p*_trend_ >0.999). Among the radiation therapy subset, EDR use grew from 7% in 2004 to 22% in 2019 (*p*_trend_ <0.0001) and median BED_10_ increased from 53 to 59 Gy (*p*_trend_ = 0.0004). Among CDR and EDR recipients with a recorded external beam RT technique, use of 3D‐conformal RT declined from 66% in 2004 to 9% in 2019 (*p*_trend_ <0.0001). Additionally, during the study period, use of stereotactic body RT (SBRT) increased from 4% in 2004 to 29% in 2019 (*p*_trend_ <0.0001), and intensity‐modulated RT (IMRT) increased from 30% in 2004 to 62% in 2019 (*p*_trend_ = 0.017). Temporal trends in utilization of RT technique are shown in Figure S1.

### 3.2 Correlates of EDR use

**FIGURE 3:**
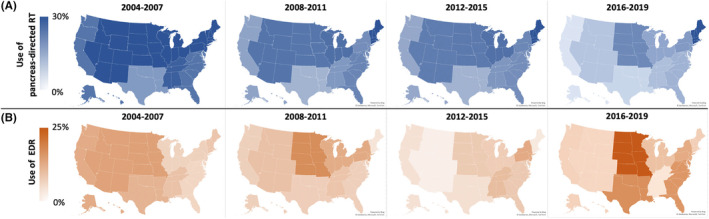
Geographic trends in utilization of pancreas‐directed RT and EDR. (A) Maps of United States regions showing fractions of patients treated with pancreas‐directed RT as a proportion of patients in the entire study cohort, stratified by 4‐year periods. (B) Maps of United States regions showing fractions of patients treated with escalated‐dose RT as a proportion of patients in the radiation therapy subset. Each set of maps was standardized to the corresponding color scales provided. EDR indicates escalated‐dose radiotherapy; LAPC, locally advanced pancreatic adenocarcinoma; RT, radiotherapy.

For the radiation therapy subset, univariate and multivariable logistic regression of factors associated with EDR use are shown in Table 2. Patients who received EDR generally presented with greater likelihood of being treated at an academic/research institution (adjusted odds ratio [aOR] 1.46, 95% CI 1.30–1.65; *p* < 0.001). Greater receipt of EDR also associated with facility location in—as compared to “New England” [Connecticut, Massachusetts, Maine, New Hampshire, Rhode Island, and Vermont]—the “West North Central” states [Iowa, Kansas, Minnesota, Missouri, North Dakota, Nebraska, and South Dakota] (aOR 2.40, 95% CI 1.80–3.20; *p* < 0.001). Traveling more than 30 miles to the treatment facility (aOR 1.56, 95% CI 1.32–1.84; *p* < 0.001) and LAPC diagnosis between 2016 and 2019 (aOR 2.54, 95% CI 2.13–3.04; *p* < 0.001) also correlated with greater EDR receipt. EDR treatment further differed from CDR in that a smaller proportion of EDR patients had a primary tumor location in the pancreas head (aOR 0.80, 95% CI 0.69–0.93; *p* = 0.003) or received chemotherapy (aOR 0.60, 95% CI 0.50–0.73; *p* < 0.001). Temporal trends in the use of pancreas‐directed RT among geographic regions of the United States are displayed as maps in Figure 3.

**TABLE 2:** Unadjusted and adjusted predictors of receipt of escalated‐dose pancreas‐directed RT for patients with unresected locally advanced pancreatic adenocarcinoma in radiation therapy subset.

Attribute	Univariate analysis			Multivariable analysis		
	Unadjusted OR	95% CI	*p* Value	Adjusted OR	95% CI	*p* Value
Age at diagnosis	1.01	1.01–1.01	<0.001^a^	1.01	1.00–1.01	0.01
Sex						
Male	1.00	Ref				
Female	1.02	0.92–1.14	0.65			
Primary site of tumor						
Body‐tail	1.00	Ref		1.00	Ref	
Head	0.82	0.71–0.93	0.003	0.80	0.69–0.93	0.003
Other	0.87	0.73–1.03	0.11	0.92	0.76–1.11	0.39
T stage						
T2	1.00	Ref		1.00	Ref	
T3‐4	0.82	0.73–0.93	0.003	1.04	0.90–1.20	0.60
N stage						
N0	1.00	Ref		1.00	Ref	
N1	0.84	0.75–0.94	0.003	0.93	0.81–1.05	0.23
Charlson‐Deyo comorbidity score						
0–1	1.00	Ref		1.00	Ref	
≥ 2	1.13	0.95–1.35	0.18	1.00	0.82–1.21	0.98
Insurance carrier						
No insurance	1.00	Ref				
Private	1.22	0.79–1.91	0.37			
Medicaid	1.17	0.71–1.93	0.53			
Medicare	1.45	0.93–2.24	0.10			
Other government	2.04	1.16–3.59	0.01			
Facility type						
Nonacademic/research	1.00	Ref		1.00	Ref	
Academic/research	1.70	1.53–1.89	<0.001^a^	1.46	1.30–1.65	<0.001^a^
Facility location						
New England	1.00	Ref		1.00	Ref	
Middle Atlantic	1.90	1.47–2.47	<0.001^a^	1.68	1.27–2.22	<0.001^a^
South Atlantic	1.68	1.30–2.17	<0.001^a^	1.66	1.27–2.18	<0.001^a^
East North Central	1.48	1.14–1.93	0.003	1.60	1.21–2.13	0.001^a^
East South Central	1.27	0.92–1.77	0.15	1.26	0.89–1.79	0.20
West North Central	2.81	2.15–3.68	<0.001^a^	2.40	1.80–3.20	<0.001^a^
West South Central	1.90	1.38–2.6	<0.001^a^	1.57	1.11–2.22	0.01
Mountain	1.18	0.81–1.71	0.38	1.34	0.90–2.00	0.15
Pacific	1.24	0.92–1.68	0.16	1.34	0.98–1.84	0.07
Patient residential setting						
Metro	1.00	Ref				
Urban	1.20	1.03–1.39	0.02			
Rural	0.89	0.60–1.32	0.56			
Distance to treatment facility, miles						
0–5	1.00	Ref		1.00	Ref	
5.1–10	1.04	0.87–1.25	0.64	0.98	0.82–1.18	0.85
10.1–30	1.18	1.00–1.38	0.05	1.09	0.93–1.29	0.29
>30	1.82	1.55–2.13	<0.001^a^	1.56	1.32–1.84	<0.001^a^
Year of diagnosis						
2004–2007	1.00	Ref		1.00	Ref	
2008–2011	1.15	0.97–1.38	0.11	1.16	0.96–1.40	0.11
2012–2015	0.91	0.76–1.09	0.32	0.88	0.72–1.06	0.17
2016–2019	2.66	2.26–3.13	<0.001^a^	2.54	2.13–3.04	<0.001^a^
Use of chemotherapy						
No	1.00	Ref		1.00	Ref	
Yes	0.56	0.48–0.66	<0.001^a^	0.60	0.50–0.73	<0.001^a^

*Note*: OR >1 indicates increased likelihood of receiving escalated‐dose RT and OR <1 decreased likelihood of receiving escalated‐dose RT.

Abbreviations: OR, odds ratio; RT, radiotherapy.

^a^ Significant at *p* < 0.0017 (Bonferroni for 30 comparisons).

### 3.3 Survival

**FIGURE 4:**
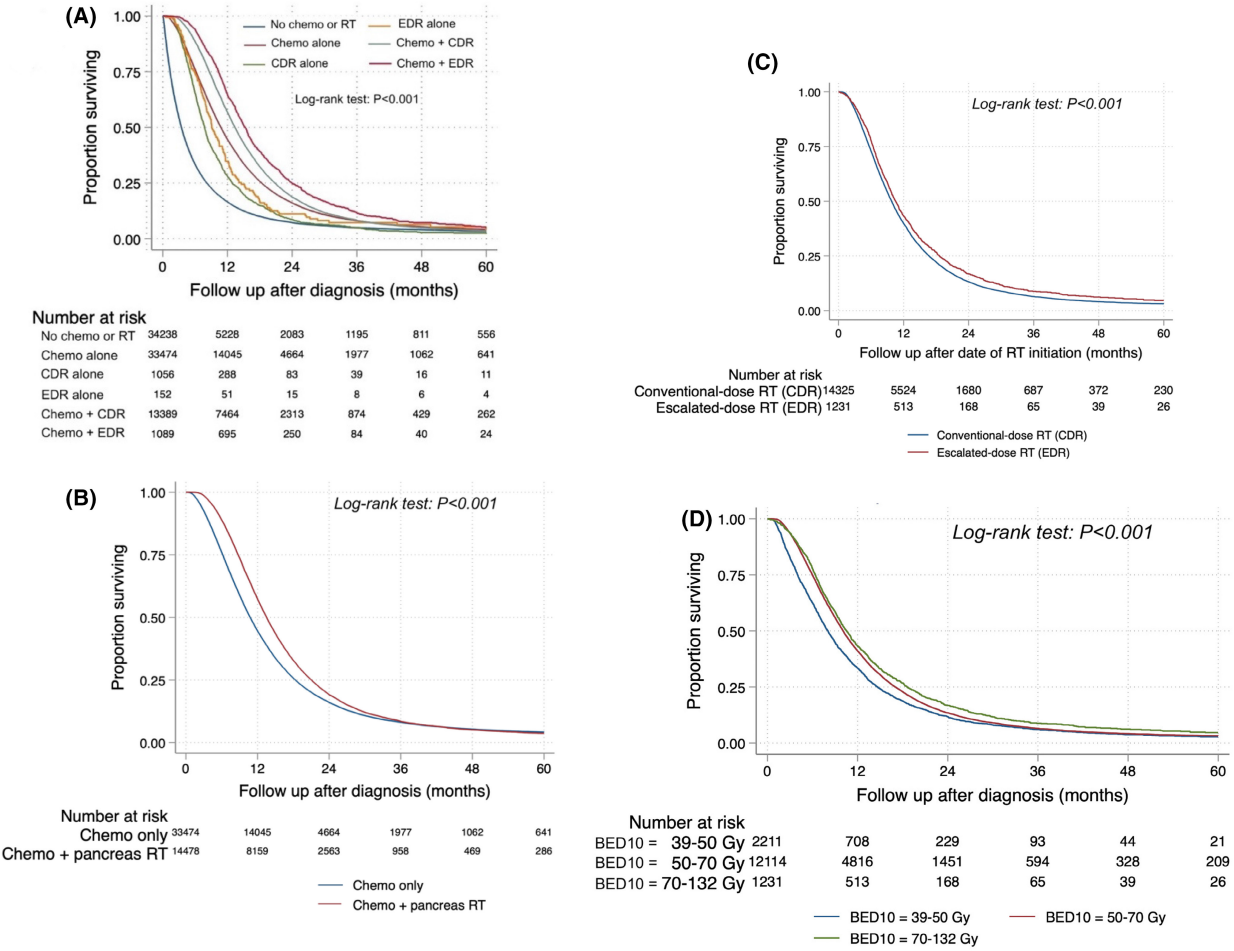
Kaplan–Meier survival estimates for (A) Patients in entire study cohort treated with combinations of chemotherapy with CDR or EDR, (B) Patients in definitive therapy subset treated with either chemotherapy alone or chemotherapy and radiation, and (C) Patients in radiation therapy subset treated with CDR alone or EDR alone, considering the time origin as date of RT initiation for landmark analysis, and (D) Patients in radiation therapy subset treated with a dose in one of three BED_10_ ranges; CDR, conventional‐dose radiotherapy; EDR, escalated‐dose radiotherapy; RT, radiotherapy.

Survival data were available for 91% (*n* = 83,398) of the entire study cohort. Survival for entire study cohort patients treated with combinations of chemotherapy with CDR or EDR are shown in Figure 4A. Among definitive therapy subset patients who received either chemotherapy alone or CT + RT (*n* = 52,780), 91% (*n* = 47,952) had available survival data, which are illustrated in Figure 4B. Survival outcomes for 92% (*n* = 15,686) of the radiation therapy subset with non‐missing data, stratified by receipt of CDR versus EDR, are displayed in Figure 4C. Survival outcomes for 85% (*n* = 14,386) of the radiation therapy subset receiving BED_10_ ≥39 and ≤50 Gy, BED_10_ >50 and ≤70 Gy, or BED_10_ >70 and ≤132 Gy are shown in Figure 4D. Univariate and multivariable associations with survival are shown in Table 3. At median follow‐up of 62.2 months (95% CI 59.7–66.0), median OS estimates were 13.0 (95% CI 12.8–13.1) months for patients receiving CDR and 14.5 (95% CI 13.8–15.2) months for patients receiving EDR.

**TABLE 3:** Unadjusted and adjusted predictors of survival for patients with unresected locally advanced pancreatic adenocarcinoma in radiation therapy subset.

Attribute	Univariate Cox regression			Multivariate Cox regression		
	Unadjusted HR	95% CI	*P‐*value	Adjusted HR	95% CI	*P‐*value
Age at diagnosis, years	1.01	1.008–1.01	<0.001^a^	1.01	1.006–1.01	<0.001^a^
Sex						
Male	1.00	Ref	Ref			
Female	0.98	0.97–1	0.085			
Primary site of tumor						
Body‐tail	1.00	Ref	Ref	1.00	Ref	Ref
Head	1.17	1.12–1.22	<0.001^a^	1.14	1.09–1.2	<0.001^a^
Other	1.06	1–1.12	0.033	1.05	0.98–1.11	0.046
T stage						
T2	1.00	Ref	Ref	1.00	Ref	Ref
T3‐4	0.93	0.89–0.97	0.001^a^	0.98	0.94–1.03	0.045
N stage						
N0	1.00	Ref	Ref	1.00	Ref	Ref
N1	1.08	1.04–1.12	<0.001^a^	1.08	1.04–1.12	<0.001^a^
Charlson‐Deyo comorbidity score						
0–1	1.00	Ref	Ref	1.00	Ref	Ref
≥ 2	1.12	1.06–1.19	<0.001^a^	1.14	1.07–1.22	0.004
Insurance carrier						
No insurance	1.00	Ref	Ref			
Private	0.82	0.72–0.92	0.001^a^			
Medicaid	0.87	0.76–1	0.056			
Medicare	0.96	0.86–1.09	0.547			
Other government	0.87	0.73–1.04	0.125			
Facility type						
Nonacademic/research	1.00	Ref	Ref	1.00	Ref	Ref
Academic/research	0.84	0.81–0.87	<0.001^a^	0.92	0.88–0.95	<0.001^a^
Facility location						
New England	1.00	Ref	Ref	1.00	Ref	Ref
Middle Atlantic	1.02	0.95–1.1	0.506	1.05	0.97–1.13	0.065
South Atlantic	1.22	1.14–1.3	<0.001^a^	1.27	1.18–1.36	<0.001^a^
East North Central	1.24	1.16–1.32	<0.001^a^	1.25	1.16–1.35	<0.001^a^
East South Central	1.32	1.21–1.44	<0.001^a^	1.38	1.26–1.52	<0.001^a^
West North Central	1.23	1.14–1.33	<0.001^a^	1.22	1.12–1.32	<0.001^a^
West South Central	1.11	1.01–1.22	0.031	1.19	1.07–1.31	0.002
Mountain	1.15	1.04–1.26	0.005	1.16	1.04–1.29	0.009
Pacific	1.10	1.02–1.19	0.013	1.07	0.99–1.17	0.022
Patient residential setting						
Metropolitan	1.00	Ref	Ref	1.00	Ref	Ref
Urban	1.12	1.07–1.17	<0.001^a^	1.06	1–1.12	0.001^a^
Rural	1.28	1.15–1.43	<0.001^a^	1.13	1–1.28	0.001^a^
Distance traveled, miles						
0–5	1.00	Ref	Ref	1.00	Ref	Ref
5.1–10	0.93	0.88–0.98	0.004	0.94	0.89–0.99	0.013
10.1–30	0.92	0.88–0.97	0.001^a^	0.97	0.92–1.01	0.007
> 30	0.91	0.86–0.95	<0.001^a^	0.95	0.89–1	0.001^a^
AJCC TNM staging edition number					Ref	Ref
6th	1.00	Ref	Ref	1.00	Ref	Ref
7th	0.72	0.69–0.74	<0.001^a^	0.85	0.8–0.91	0.001^a^
8th	0.54	0.5–0.57	<0.001^a^	0.81	0.72–0.9	0.001^a^
Year of diagnosis						
2004–2007	1.00	Ref	Ref	1.00	Ref	Ref
2008–2011	0.92	0.88–0.96	<0.001^a^	1.00	0.95–1.07	0.013
2012–2015	0.70	0.67–0.73	<0.001^a^	0.84	0.77–0.91	0.007
2016–2019	0.55	0.52–0.58	<0.001^a^	0.66	0.6–0.73	0.001^a^
Use of chemotherapy						
No	1.00	Ref	Ref	1.00	Ref	Ref
Yes	0.55	0.52–0.59	<0.001^a^	0.59	0.55–0.63	<0.001^a^
Dose of RT						
Conventional (39 Gy ≤ BED ≤70 Gy)	1.00	Ref	Ref	1.00	Ref	Ref
Escalated (70 Gy < BED ≤132 Gy)	0.84	0.79–0.9	<0.001^a^	0.85	0.8–0.91	<0.001^a^

*Note*: HR >1 indicates higher hazard of death and HR <1 lower hazard of death.

Abbreviations: HR, hazard ratio; RT, radiotherapy.

^a^ Significant at *p* < 0.0017 (Bonferroni for 30 comparisons).

In multivariable Cox regression of radiation therapy subset patients with data available (*n* = 13,579), older age (adjusted hazard ratio [aHR] 1.01, 95% CI 1.006–1.010; *p* < 0.001), N1 versus N0 disease (aHR 1.08, CI 1.04–1.12; *p* < 0.001), and patient residence in a rural versus metropolitan setting (aHR 1.13, 95% CI 1.00–1.28; *p* = 0.001) correlated with shorter OS. On the contrary, treatment at an academic/research facility (aHR 0.92, 95% CI 0.88–0.95; *p* < 0.001), receipt of chemotherapy (aHR 0.59, 95% CI 0.55–0.63; *p* < 0.001), and receipt of EDR versus CDR (aHR 0.85, 95% CI 0.80–0.91; *p* < 0.001) correlated with longer OS.

We also performed a landmark analysis of survival with origin at the date of RT initiation. For 13,458 CT + RT patients with available data on the number of days before initiation of pancreas‐directed RT, multivariable Cox regression demonstrated that higher RT dose, as both a dichotomous variable (EDR vs. CDR; aHR 0.88, 95% CI 0.83–0.95; *p* < 0.001) and continuous variable (BED_10_; aHR 0.993, 95% CI 0.991–0.995; *p* < 0.001), was associated with a lower hazard of death. Survival curves considering the conditional landmark are shown in Figure 4C with median OS estimates for CDR and EDR of 9.8 (95% CI 9.6–9.9) months and 10.5 (95% CI 10.0–11.1) months, respectively.

Multivariable analysis using the conditional landmark (Table S1) correlated older age (aHR 1.01, 95% CI 1.005–1.01; *p* < 0.001), N1 versus N0 disease (aHR 1.07, CI 1.03–1.11; *p* < 0.001), and Charlson‐Deyo comorbidity score of 2 or greater versus a score of 0–1 (aHR 1.14, 95% CI 1.05–1.20; *p* < 0.001) with shorter OS. On the contrary, treatment at an academic/research facility (aHR 0.93, 95% CI 0.89–0.96; *p* < 0.001) and receipt of EDR versus CDR (aHR 0.88, 95% CI 0.83–0.95; *p* < 0.001) correlated with longer OS.

Using the conditional landmark, multivariable Cox regression results were also conducted for a subset of 6236 CT + RT patients who received multi‐agent chemotherapy (Table S2). Notably, a significant association between EDR versus CDR was no longer observed (aHR 0.92, 95% CI 0.83–1.01; *p* = 0.094) in this subset analysis.

## 4 DISCUSSION

The use of EDR following upfront chemotherapy for the treatment of LAPC is controversial given a lack of supporting randomized data. While large database analyses have provided some evidence that higher RT doses may be associated with improved survival,^20^, ^21^, ^22^ this study provides one the first contemporary examinations of utilization of pancreas‐directed RT and of EDR in particular. Through this study, we showed that there has been an increase in EDR use as a proportion of patients receiving pancreatic RT (approximately 22% in 2019) but no increase in utilization of pancreatic RT overall for patients with LAPC. We found that treatment at an academic/research facility and treatment in 2016–2019 associated with greater utilization of EDR, whereas use of chemotherapy associated with less utilization. We additionally demonstrated that utilization of highly conformal delivery techniques, including IMRT and SBRT, has increased dramatically over the study period. Lastly, we showed that use of EDR is associated with longer survival versus CDR, even after adjustment for clinicopathologic and treatment‐related factors.

Three randomized trials examining the role of pancreas‐directed RT in patients with LAPC were published during the study period, potentially influencing RT utilization: the Fédération Francophone de Cancérologie Digestive and Société Française de Radiothérapie Oncologique (FFCD–SFRO) study published in 2008,^23^ the Eastern Cooperative Oncology Group (ECOG) 4201 trial published in 2011,^24^ and the LAP‐07 trial, presented as an abstract in 2013 and as a full manuscript in 2016.^7^ These studies provide conflicting evidence regarding the benefits of RT in patients with LAPC. The ECOG trial, which compared gemcitabine with or without RT to a dose of 50.4 Gy in 28 fractions (BED_10_ 59.5 Gy) using 2D/3D‐conformal technique, showed that the RT arm had longer survival (median 11.1 months vs. 9.2 months), but with a greater incidence of grade 4 toxicities. Conversely, LAP‐07 used a 2 × 2 factorial design to compare patients receiving upfront gemcitabine with or without erlotinib, followed by chemotherapy or chemoradiotherapy to a dose of 54 Gy in 30 fractions (BED_10_ 63.7 Gy) using 3D‐conformal radiation. This study showed no overall survival advantage to the addition of RT (median 15.2 vs. 16.5 months) but did report better local control and longer chemotherapy‐free interval compared to chemotherapy alone. Though it is difficult to draw direct correlations between trial results and utilization of pancreas‐directed RT, the negative overall survival findings of the LAP‐07 trial appear to coincide temporally with a nominal decrease in pancreas‐directed RT. It is notable that none of these studies utilized FOLFIRINOX or gemcitabine/nab‐paclitaxel chemotherapy nor did they use IMRT or SBRT, which better enable safe dose‐escalation of RT to the tumor while meeting normal tissue constraints. Despite the negative trial findings, the use of EDR has increased particularly since 2016, coinciding with greater use of multi‐agent chemotherapies such as FOLFIRINOX. More recent studies have also shown that dose escalation magnetic resonance‐guided radiation associates with longer survival.^14^, ^25^, ^26^

Randomized evidence regarding RT dose escalation is lacking. RTOG 1201 was a phase II randomized trial comparing CDR or EDR with concurrent gemcitabine/nab‐paclitaxel to gemcitabine/nab‐paclitaxel alone for patients with LAPC, but it was closed early due to poor accrual. Nevertheless, several retrospective studies have provided evidence of the benefits of EDR. An NCDB analysis examined the role of dose escalation for LAPC and showed an OS benefit to doses escalated above 40 Gy.^20^ However, this study notably considered patients treated between 1998 and 2002, well before the popularization of more conformal RT techniques. One study from MD Anderson Cancer Center showed that patients receiving EDR with IMRT in 15 to 28 fractions (67.5–70 Gy; BED_10_ >70 Gy) had improved OS, local‐ and regional‐recurrence‐free survival, and fewer gastrointestinal and overall toxicities when compared with those receiving CDR.^13^, ^27^ Similarly, a study from Memorial Sloan Kettering Cancer Center of 119 patients with LAPC considered patients treated with modern RT techniques to 75 Gy in 25 fractions (BED_10_ 97.5 Gy) or 67.5 Gy in 15 fractions (BED_10_ 97.9 Gy) and showed favorable results: a median OS of 26.8 months from diagnosis and 18.4 months from EDR.^12^ Ongoing studies seek to examine the role of EDR in the context of immunotherapy and/or targeted therapies, which will add to our understanding of the role of dose escalation in patients with LAPC.^28^

We did not observe a significant change in the use of pancreas‐directed RT over the study period, but EDR use as a proportion of pancreas‐directed RT increased significantly after 2015, from 7% in 2015 to 22% in 2019. This observation may be driven by the widespread availability of conformal treatment methods, image guidance, and motion management techniques, as well as greater belief among treating radiation oncologists in the benefits of EDR versus CDR. A possible contributor to these findings is the publication of a seminal phase 2 study in 2015 showing favorable survival and toxicity among LAPC patients receiving gemcitabine and SBRT, which may have boosted enthusiasm among radiation oncologists for EDR regimens, leading to a greater proportion of patients receiving EDR despite dampened interest in the use of pancreas‐directed RT for LAPC as a whole.^29^ Among patients receiving pancreas‐directed RT, use of 3D‐CRT declined by more than six‐fold over the 16‐year study period, while use of SBRT and IMRT together accounted for approximately 90% of patients by 2019. The SMART trial utilizes magnetic resonance‐guided on‐table adapted radiation therapy, real‐time soft tissue tracking, and radiation beam gating in an effort to reduce gastrointestinal toxicities for patients with borderline resectable or inoperable LAPC.^26^ Recently published data from the SMART trial have shown favorable outcomes, including low rates of grade ≥3 gastrointestinal toxicities and a high rate of 2‐year OS, warranting further investigation in this patient population.^25^, ^30^, ^31^

Factors associated with greater use of EDR included treatment at an academic/research facility and treatment in 2016–2019, which may reflect the growing availability of technologies to enable dose escalation at such centers. Additionally, no receipt of chemotherapy was associated with greater use of EDR. It is likely that EDR in these cases was preferentially used by radiation oncologists as a form of treatment intensification for patients unable to receive standard‐of‐care chemotherapy. We observed significant regional variations possibly reflecting institutional practice protocols and/or physician training biases.

This study has several limitations. Population‐based observational studies have shown poor agreement with randomized trials, potentially limiting the value of survival HR estimates in this and similar studies.^32^ While associations between geographical location and distance traveled with survival were identified, they are possibly explained by residual confounding from other case mix factors including clinical and sociodemographic factors. It was also not possible through the current study to identify drivers of these findings or strategies to mitigate potential disparities. Similarly, the association of pancreatic head tumor location with shorter survival in the current study runs counter to other studies that have shown more favorable long‐term prognosis with tumors of the pancreatic head, also suggesting residual confounding in the analyses presented.^33^ Over the study period, AJCC 6th, 7th, and 8th edition staging systems were used, resulting in a potentially heterogeneous study population and source of bias. In particular, the 6th and 7th editions did not utilize a minimum size requirement nor was there an N2 classification. As a result, patients with higher risk disease that were excluded in the 8th edition era may have not been excluded in previous iterations. Though we have attempted to account for this source of bias by including AJCC staging edition in the multivariable model, there is nevertheless potential for bias and confounding. RT to regional lymph nodes may be an important consideration in achieving more durable locoregional control.^34^ However, we were unable to robustly analyze the role of nodal irradiation in the present analysis using the NCDB. Precise tumor location details with details regarding regional anatomy—such as the presence of duodenal invasion, a contraindication to RT dose escalation—are not available in the NCDB, likely leading to selection bias given that patients with unfavorable anatomy would not have been candidates for dose escalation to begin with. Another possible contributor to selection bias is the lack of recorded pretherapy treatment intent in the NCDB; it is unknown if the nonsurgically managed patients in this study were deemed unresectable upfront or after preoperative‐intent chemotherapy. It is also possible that, compared to patients who received CDR, those who received EDR more frequently went on to receive resection, which is typically associated with longer survival. Given that patients who received surgery were excluded from our analysis, such a phenomenon may have led us to underestimate the potential survival benefit associated with EDR. Specific chemotherapy agents are not recorded in the NCDB, making it difficult to understand if the benefits of dose escalation were limited to a chemotherapy subgroup as the standard of care evolved over the study period. While the association between EDR and longer survival was no longer observed when considering the subset of patients who received multiagent chemotherapy, it is difficult to know if this is due to small sample size in the setting of a small observed effect size, or if the association of EDR with longer survival is attenuated by the use of more efficacious chemotherapy. RT details such as pretherapy imaging and motion management were not included in the NCDB. The linear‐quadratic model was utilized to calculate BED_10_ in the present analysis, but its application to hypofractionated RT regimens remains controversial.^35^ The NCDB lacks metrics of response to treatment such as local control, which limits our ability to evaluate causality for the association between EDR and longer survival. Toxicities are also not recorded and are of particular importance for patients being treated with EDR; we are unable to accurately evaluate the therapeutic ratio of EDR from the NCDB without this information. The theoretical upper limit of the proportion of patients eligible for EDR is not known and cannot be determined from data in NCDB. Conformal radiotherapy techniques, image guidance, and motion management significantly evolved over the study period—all of which increase the potential to safely administer RT as a possible conduit to more durable local control. However, more widespread utilization of EDR is contingent on the publication of further randomized data demonstrating a patient‐centered benefit.

To conclude, the results of this study show that use of EDR for patients with unresected LAPC is increasing as a proportion of those receiving pancreas‐directed RT despite steady utilization of pancreas‐directed RT overall. In addition, EDR may be associated with longer survival when compared with CDR, but randomized trials incorporating modern chemotherapy and EDR as well as continued real‐world data are needed to better establish this association. The effect size of EDR that we observed in the current, utilization trends of pancreas‐directed RT in the United States, and lessons from prior efforts such as RTOG 1201 may help inform the design of future randomized trials for LAPC.

## AUTHOR CONTRIBUTIONS

**Christopher Shi:** Conceptualization (supporting); formal analysis (lead); investigation (equal); methodology (supporting); project administration (supporting); visualization (lead); writing – original draft (equal); writing – review and editing (equal). **Brian De:** Conceptualization (equal); data curation (equal); formal analysis (supporting); investigation (supporting); methodology (equal); project administration (equal); resources (supporting); supervision (equal); visualization (supporting); writing – original draft (equal); writing – review and editing (equal). **Hop S. Tran Cao:** Conceptualization (supporting); data curation (lead); methodology (supporting); resources (supporting); writing – review and editing (supporting). **Suyu Liu:** Formal analysis (supporting). **Marcus A. Florez:** Formal analysis (supporting); writing – review and editing (supporting). **Ramez Kouzy:** Writing – review and editing (supporting). **Adam J. Grippin:** Writing – review and editing (supporting). **Matthew H. G. Katz:** Writing – review and editing (supporting). **Ching‐Wei D. Tzeng:** Writing – review and editing (supporting). **Naruhiko Ikoma:** Writing – review and editing (supporting). **Michael P. Kim:** Writing – review and editing (supporting). **Sunyoung Lee:** Writing – review and editing (supporting). **Jason Willis:** Writing – review and editing (supporting). **Sonal S. Noticewala:** Writing – review and editing (supporting). **Bruce D. Minsky:** Writing – review and editing (supporting). **Grace L. Smith:** Writing – review and editing (supporting). **Emma B. Holliday:** Writing – review and editing (supporting). **Cullen M. Taniguchi:** Writing – review and editing (supporting). **Albert C. Koong:** Conceptualization (supporting); writing – review and editing (supporting). **Prajnan Das:** Conceptualization (supporting); writing – review and editing (supporting). **Ethan B. Ludmir:** Conceptualization (equal); data curation (supporting); formal analysis (supporting); funding acquisition (supporting); methodology (equal); project administration (equal); resources (supporting); supervision (equal); writing – original draft (supporting); writing – review and editing (supporting). **Eugene J. Koay:** Conceptualization (equal); data curation (supporting); funding acquisition (equal); methodology (equal); project administration (equal); supervision (equal); writing – original draft (supporting); writing – review and editing (supporting).

## CONFLICT OF INTEREST STATEMENT

BD reports honoraria from Sermo, Inc. PD reports honoraria from ASTRO, ASCO, Imedex, and Bayer. CT reports a consulting/advisory role with Accuray. EJK reports grants from National Institutes of Health, Stand Up 2 Cancer, MD Anderson Cancer Center, Philips Healthcare, Elekta, and GE Healthcare; personal fees from RenovoRx and Taylor and Francis; and a consulting/advisory role with Augmenix. ACK reports ownership of shares in Aravive, Inc. All reported conflicts are outside of the submitted work.

## ETHICS STATEMENT

This study protocol was reviewed and approved by the MD Anderson Institutional Review Board, approval number 2020–0512. The requirement for informed consent was waived.

## Supporting information


Appendix S1.

## Data Availability

The primary dataset (National Cancer Database) is publicly available through the American College of Surgeons (https://www.facs.org/quality‐programs/cancer/ncdb). The specific statistical analyses used during the current study are available from the corresponding author on reasonable request within 1 year of publication.
