# Supplementary material for: Escalated‐dose radiotherapy for unresected locally advanced pancreatic cancer: Patterns of care and survival in the United States
Source: Cancer Med. 2024 Jun 26;13(12):e7434. doi: 10.1002/cam4.7434 (PMC11200087; doi:10.1002/cam4.7434)
Supplement: Supplementary file 1 — Appendix S1. [file CAM4-13-e7434-s001.docx]

**SUPPLEMENTARY MATERIAL**


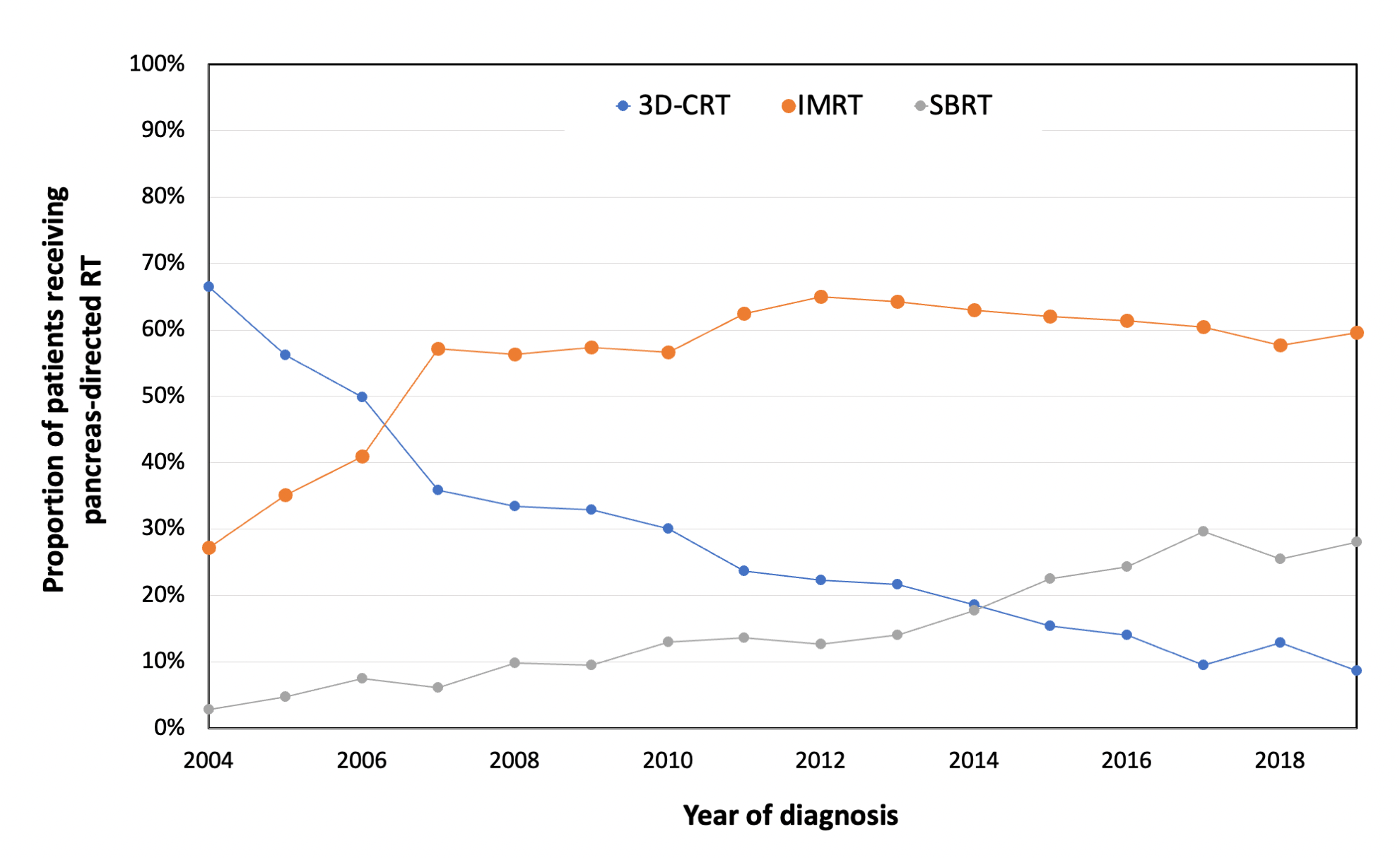


**Supplementary Figure 1**. Utilization of pancreas-directed radiotherapy, stratified by radiotherapy technique, among included LAPC patients in the radiation therapy subset. Abbreviations: 3D-CRT, 3D-conformal radiotherapy; IMRT, intensity-modulated radiotherapy; LAPC, locally advanced pancreatic adenocarcinoma; RT, radiotherapy; SBRT, stereotactic body radiotherapy.

|  | **Univariate Cox regression** | | | **Multivariable Cox regression** | | |
| --- | --- | --- | --- | --- | --- | --- |
| **Attribute** | **Unadjusted HR** | **95% CI** | ***P-*value** | **Adjusted HR** | **95% CI** | ***P-*value** |
| Age at diagnosis, years | 1.01 | 1.007-1.01 | <0.001^a^ | 1.01 | 1.005-1.01 | <0.001^a^ |
| Sex |  |  |  |  |  |  |
| Male | 1.00 | Ref | Ref |  |  |  |
| Female | 0.96 | 0.93-0.99 | 0.013 |  |  |  |
| Primary site of tumor |  |  |  |  |  |  |
| Body-tail | 1.00 | Ref | Ref | 1.00 | Ref | Ref |
| Head | 1.17 | 1.12-1.22 | <0.001^a^ | 1.16 | 1.11-1.22 | <0.001^a^ |
| Other | 1.07 | 1.01-1.13 | 0.021 | 1.06 | 1-1.13 | 0.049 |
| T stage |  |  |  |  |  |  |
| T2 | 1.00 | Ref | Ref | 1.00 | Ref | Ref |
| T3-4 | 0.92 | 0.89-0.96 | <0.001^a^ | 0.993 | 0.95-1.04 | 0.758 |
| N stage |  |  |  |  |  |  |
| N0 | 1.00 | Ref | Ref | 1.00 | Ref | Ref |
| N1 | 1.07 | 1.04-1.12 | <0.001^a^ | 1.07 | 1.03-1.11 | <0.001^a^ |
| Charlson-Deyo comorbidity score |  |  |  |  |  |  |
| 0-1 | 1.00 | Ref | Ref | 1.00 | Ref | Ref |
| ≥2 | 1.14 | 1.05-1.20 | <0.001^a^ | 1.14 | 1.05-1.20 | <0.001^a^ |
| Insurance carrier |  |  |  |  |  |  |
| No insurance | 1.00 | Ref | Ref |  |  |  |
| Private | 0.82 | 0.72-0.92 | 0.001^a^ |  |  |  |
| Medicaid | 0.88 | 0.77-1.01 | 0.069 |  |  |  |
| Medicare | 0.96 | 0.85-1.08 | 0.503 |  |  |  |
| Other government | 0.89 | 0.74-1.06 | 0.192 |  |  |  |
| Facility type |  |  |  |  |  |  |
| Nonacademic/research | 1.00 | Ref | Ref | 1.00 | Ref | Ref |
| Academic/research | 0.87 | 0.84-0.9 | 0.001^a^ | 0.93 | 0.89-0.96 | <0.001^a^ |
| Facility location |  |  |  |  |  |  |
| New England | 1.00 | Ref | Ref | 1.00 | Ref | Ref |
| Middle Atlantic | 1.04 | 0.97-1.12 | 0.289 | 1.06 | 0.99-1.15 | 0.105 |
| South Atlantic | 1.20 | 1.12-1.29 | <0.001^a^ | 1.24 | 1.16-1.34 | <0.001^a^ |
| East North Central | 1.19 | 1.11-1.28 | <0.001^a^ | 1.21 | 1.13-1.31 | <0.001^a^ |
| East South Central | 1.27 | 1.16-1.38 | <0.001^a^ | 1.32 | 1.21-1.45 | <0.001^a^ |
| West North Central | 1.20 | 1.11-1.3 | <0.001^a^ | 1.19 | 1.1-1.29 | <0.001^a^ |
| West South Central | 1.12 | 1.02-1.23 | 0.015 | 1.18 | 1.07-1.3 | 0.001^a^ |
| Mountain | 1.15 | 1.05-1.27 | 0.004 | 1.16 | 1.04-1.29 | 0.006 |
| Pacific | 1.14 | 1.06-1.24 | 0.001^a^ | 1.12 | 1.03-1.22 | 0.008 |
| Patient residential setting |  |  |  |  |  |  |
| Metropolitan | 1.00 | Ref | Ref | 1.00 | Ref | Ref |
| Urban | 1.09 | 1.04-1.14 | <0.001^a^ | 1.05 | 0.99-1.11 | 0.108 |
| Rural | 1.21 | 1.08-1.35 | 0.001^a^ | 1.11 | 0.98-1.25 | 0.114 |
| Distance travelled, miles |  |  |  |  |  |  |
| 0-5 | 1.00 | Ref | Ref | 1.00 | Ref | Ref |
| 5.1-10 | 0.94 | 0.89-0.98 | 0.010 | 0.95 | 0.9-1 | 0.037 |
| 10.1-30 | 0.95 | 0.91-0.995 | 0.030 | 0.98 | 0.93-1.03 | 0.361 |
| >30 | 0.93 | 0.88-0.97 | 0.002 | 0.95 | 0.9-1.008 | 0.093 |
| AJCC TNM staging edition number |  |  |  |  |  |  |
| 6th | 1.00 | Ref | Ref | 1.00 | Ref | Ref |
| 7th | 0.8219359 | 0.79-0.85 | <0.001^a^ | 0.89 | 0.84-0.95 | 0.001^a^ |
| 8th | 0.7172836 | 0.67-0.77 | <0.001^a^ | 0.90 | 0.8-1.01 | 0.082 |
| Year of diagnosis |  |  |  |  |  |  |
| 2004-2007 | 1.00 | Ref | Ref | 1.00 | Ref | Ref |
| 2008-2011 | 0.98 | 0.93-1.02 | 0.329 | 1.04 | 0.98-1.1 | 0.250 |
| 2012-2015 | 0.82 | 0.78-0.85 | <0.001^a^ | 0.93 | 0.86-1.01 | 0.081 |
| 2016-2019 | 0.72 | 0.68-0.76 | <0.001^a^ | 0.80 | 0.72-0.88 | <0.001^a^ |
| Use of chemotherapy |  |  |  |  |  |  |
| No | 1.00 | Ref | Ref | 1.00 | Ref | Ref |
| Yes | 0.63 | 0.59-0.67 | <0.001^a^ | 0.65 | 0.61-0.7 | <0.001^a^ |
| Dose of RT |  |  |  |  |  |  |
| Conventional (39 Gy ≤ BED ≤ 70 Gy) | 1.00 | Ref | Ref | 1.00 | Ref | Ref |
| Escalated (70 Gy < BED ≤ 132 Gy) | 0.88 | 0.83-0.94 | <0.001^a^ | 0.88 | 0.83-0.95 | <0.001^a^ |

**Supplementary Table 1.** Unadjusted and adjusted predictors of survival for patients with unresected locally advanced pancreatic adenocarcinoma receiving chemotherapy and RT or RT alone, using date of radiotherapy initiation as origin of landmark analysis

Abbreviations: HR, hazard ratio; RT, radiotherapy.

HR > 1 indicates higher hazard of death and HR<1 lower hazard of death.

^a^ Significant at *P* < 0.0017 (Bonferroni for 30 comparisons).

|  | **Univariate Cox regression** | | | **Multivariable Cox regression** | | |
| --- | --- | --- | --- | --- | --- | --- |
| **Attribute** | **Unadjusted HR** | **95% CI** | ***P-*value** | **Adjusted HR** | **95% CI** | ***P-*value** |
| Age at diagnosis, years | 1.00 | 1.002-1.007 | <0.001^a^ | 1.01 | 1.003-1.01 | <0.001^a^ |
| Sex |  |  |  |  |  |  |
| Male | 1.00 | Ref | Ref |  |  |  |
| Female | 0.96 | 0.93-0.99 | 0.013 |  |  |  |
| Primary site of tumor |  |  |  |  |  |  |
| Body-tail | 1.00 | Ref | Ref | 1.00 | Ref | Ref |
| Head | 1.19 | 1.11-1.26 | <0.001^a^ | 1.18 | 1.1-1.26 | <0.001^a^ |
| Other | 1.09 | 1-1.18 | 0.039 | 1.06 | 0.974-1.16 | 0.170 |
| T stage |  |  |  |  |  |  |
| T2 | 1.00 | Ref | Ref | 1.00 | Ref | Ref |
| T3-4 | 0.95 | 0.89-1.02 | 0.167 | 0.980 | 0.91-1.06 | 0.609 |
| N stage |  |  |  |  |  |  |
| N0 | 1.00 | Ref | Ref | 1.00 | Ref | Ref |
| N1 | 1.09 | 1.04-1.12 | 0.001^a^ | 1.07 | 1.02-1.14 | 0.012 |
| Charlson-Deyo comorbidity score |  |  |  |  |  |  |
| 0-1 | 1.00 | Ref | Ref | 1.00 | Ref | Ref |
| ≥2 | 1.09 | 1.05-1.20 | 0.049 | 1.13 | 1.05-1.20 | 0.015 |
| Insurance carrier |  |  |  |  |  |  |
| No insurance | 1.00 | Ref | Ref |  |  |  |
| Private | 0.81 | 0.67-0.97 | 0.024 |  |  |  |
| Medicaid | 0.83 | 0.67-1.03 | 0.087 |  |  |  |
| Medicare | 0.91 | 0.75-1.09 | 0.291 |  |  |  |
| Other government | 0.81 | 0.62-1.06 | 0.123 |  |  |  |
| Facility type |  |  |  |  |  |  |
| Nonacademic/research | 1.00 | Ref | Ref | 1.00 | Ref | Ref |
| Academic/research | 0.90 | 0.85-0.94 | <0.001^a^ | 0.95 | 0.89-1 | 0.063 |
| Facility location |  |  |  |  |  |  |
| New England | 1.00 | Ref | Ref | 1.00 | Ref | Ref |
| Middle Atlantic | 1.07 | 0.96-1.18 | 0.216 | 1.06 | 0.95-1.18 | 0.332 |
| South Atlantic | 1.23 | 1.11-1.36 | <0.001^a^ | 1.22 | 1.1-1.37 | <0.001^a^ |
| East North Central | 1.27 | 1.15-1.41 | <0.001^a^ | 1.29 | 1.15-1.44 | <0.001^a^ |
| East South Central | 1.25 | 1.09-1.42 | 0.001^a^ | 1.30 | 1.12-1.5 | <0.001^a^ |
| West North Central | 1.32 | 1.18-1.48 | <0.001^a^ | 1.30 | 1.15-1.48 | <0.001^a^ |
| West South Central | 1.13 | 0.98-1.3 | 0.086 | 1.17 | 1-1.36 | 0.044 |
| Mountain | 1.23 | 1.07-1.41 | 0.003 | 1.22 | 1.05-1.42 | 0.011 |
| Pacific | 1.20 | 1.07-1.35 | 0.002 | 1.16 | 1.03-1.31 | 0.017 |
| Patient residential setting |  |  |  |  |  |  |
| Metropolitan | 1.00 | Ref | Ref | 1.00 | Ref | Ref |
| Urban | 1.07 | 0.99-1.15 | 0.073 | 1.02 | 0.93-1.11 | 0.651 |
| Rural | 1.44 | 1.2-1.73 | <0.001^a^ | 1.30 | 1.06-1.6 | 0.012 |
| Distance travelled, miles |  |  |  |  |  |  |
| 0-5 | 1.00 | Ref | Ref | 1.00 | Ref | Ref |
| 5.1-10 | 1.01 | 0.93-1.09 | 0.884 | 1.02 | 0.94-1.11 | 0.58 |
| 10.1-30 | 0.96 | 0.89-1.03 | 0.213 | 1.00 | 0.93-1.07 | 0.910 |
| >30 | 0.97 | 0.9-1.04 | 0.386 | 0.99 | 0.9-1.076 | 0.754 |
| AJCC TNM staging edition number |  |  |  |  |  |  |
| 6th | 1.00 | Ref | Ref | 1.00 | Ref | Ref |
| 7th | 0.81 | 0.77-0.86 | <0.001^a^ | 0.90 | 0.8-1.01 | 0.064 |
| 8th | 0.76 | 0.69-0.83 | <0.001^a^ | 0.94 | 0.8-1.11 | 0.485 |
| Year of diagnosis |  |  |  |  |  |  |
| 2004-2007 | 1.00 | Ref | Ref | 1.00 | Ref | Ref |
| 2008-2011 | 1.01 | 0.93-1.1 | 0.745 | 1.08 | 0.96-1.21 | 0.186 |
| 2012-2015 | 0.84 | 0.78-0.9 | <0.001^a^ | 0.95 | 0.82-1.1 | 0.489 |
| 2016-2019 | 0.76 | 0.7-0.83 | <0.001^a^ | 0.82 | 0.7-0.96 | 0.015 |
| Use of chemotherapy |  |  |  |  |  |  |
| No | 1.00 | Ref | Ref |  |  |  |
| Yes | 0.63 | 0.59-0.67 | <0.001^a^ |  |  |  |
| Dose of RT |  |  |  |  |  |  |
| Conventional (39 Gy ≤ BED ≤ 70 Gy) | 1.00 | Ref | Ref | 1.00 | Ref | Ref |
| Escalated (70 Gy < BED ≤ 132 Gy) | 0.91 | 0.83-0.99 | 0.023 | 0.92 | 0.83-1.01 | 0.094 |

**Supplementary Table 2.** Unadjusted and adjusted predictors of survival for patients with unresected locally advanced pancreatic adenocarcinoma receiving multi-agent chemotherapy, using date of radiotherapy initiation as origin of landmark analysis

Abbreviations: HR, hazard ratio; RT, radiotherapy.

HR > 1 indicates higher hazard of death and HR<1 lower hazard of death.

^a^ Significant at *P* < 0.0017 (Bonferroni for 30 comparisons).
